# Generation of Distinct Differentially Culturable Forms of *Burkholderia* following Starvation at Low Temperature

**DOI:** 10.1128/spectrum.02110-21

**Published:** 2022-01-05

**Authors:** Joss M. Auty, Christopher H. Jenkins, Jennifer Hincks, Anna A. Straatman-Iwanowska, Natalie Allcock, Obolbek Turapov, Edouard E. Galyov, Sarah V. Harding, Galina V. Mukamolova

**Affiliations:** a Department of Respiratory Sciences, University of Leicester, Leicester, United Kingdom; b Defence Science and Technology Laboratory, Chemical, Biological and Radiological Division, Porton Down, Salisbury, Wiltshire, United Kingdom; c FACS Facility Core Biotechnology Services, University of Leicester, Leicester, United Kingdom; d Electron Microscopy Facility, Core Biotechnology Services, University of Leicester, Leicester, United Kingdom; e Department of Genetics and Genome Biology, University of Leicester, Leicester, United Kingdom; Emory University School of Medicine

**Keywords:** *Burkholderia pseudomallei*, *Burkholderia thailandensis*, differentially culturable cells, lytic transglycosylase, coccoid cells, resuscitation

## Abstract

Bacteria have developed unique mechanisms to adapt to environmental stresses and challenges of the immune system. Here, we report that Burkholderia pseudomallei, the causative agent of melioidosis, and its laboratory surrogate, Burkholderia thailandensis, utilize distinct mechanisms for surviving starvation at different incubation temperatures. At 21°C, *Burkholderia* are present as short rods which can rapidly reactivate and form colonies on solid media. At 4°C, *Burkholderia* convert into coccoid forms that cannot be cultured on solid agar but can be resuscitated in liquid media supplemented with supernatant obtained from logarithmic phase cultures of B. thailandensis, or catalase and Tween 80, thus displaying characteristics of differentially culturable bacteria (DCB). These DCB have low intensity fluorescence when stained with SYTO 9, have an intact cell membrane (propidium iodide negative), and contain 16S rRNA at levels comparable with growing cells. We also present evidence that lytic transglycosylases, a family of peptidoglycan-remodeling enzymes, are involved in the generation of coccoid forms and their resuscitation to actively growing cells. A B. pseudomallei Δ*ltgGCFD* mutant with four *ltg* genes deleted did not produce coccoid forms at 4°C and could not be resuscitated in the liquid media evaluated. Our findings provide insights into the adaptation of *Burkholderia* to nutrient limitation and the generation of differentially culturable bacteria.

**IMPORTANCE** Bacterial pathogens exhibit physiologically distinct forms that enable their survival in an infected host, the environment and following exposure to antimicrobial agents. B. pseudomallei causes the disease melioidosis, which has a high mortality rate and is difficult to treat with antibiotics. The bacterium is endemic to several countries and detected in high abundance in the environment. Here, we report that during starvation at low temperature, B. pseudomallei produces coccoid forms that cannot grow in standard media and which, therefore, can be challenging to detect using common tools. We provide evidence that the formation of these cocci is mediated by cell wall-specialized enzymes and lytic transglycosylases, and that resuscitation of these forms occurs following the addition of catalase and Tween 80. Our findings have important implications for the disease control and detection of B. pseudomallei, an agent of both public health and defense interest.

## INTRODUCTION

The *Burkholderia* genus is a versatile group of Gram-negative bacterial species which play an important role as human, animal, and plant pathogens and inhabit complex soil communities (1). Burkholderia pseudomallei is an environmental saprophyte and the causative agent of melioidosis, an infectious disease that claims an estimated 89,000 lives annually (2). Melioidosis is endemic to 45 countries, has a high mortality (above 50% in some cases [3], often due to misdiagnosis), and is naturally resistant to many antibiotics. There is also currently no vaccine available (2). Latent infections of B. pseudomallei are also well documented and may contribute to the distribution of this pathogen in the human population (4). Recent studies detailed the detection of B. pseudomallei in nontropical countries with temperate climates (5–7), suggesting that these bacteria can survive in a wide range of temperatures.

B. pseudomallei infections are usually related to environmental exposure, highlighting the remarkable ability of this pathogen to survive in harsh environmental conditions. Of particular interest is the high prevalence of B. pseudomallei in nutrient-depleted soil, which is often found in paddy rice fields (8). However, the precise mechanisms that enable this organism to survive in such hostile conditions are poorly characterized. *Burkholderia* produce a variety of physiologically distinct forms ranging from persisters (9) to viable but nonculturable cells (VBNC) (10). VBNC are bacteria that temporarily lose the ability to produce colonies on solid agar but retain cellular integrity and the ability to resuscitate in specific conditions (11). The term “VBNC” has been disputed (12) and is still widely used to describe this phenomenon (13). Alternative definitions have been proposed and include “transiently culturable” bacteria (14), “nonculturable” bacteria (15), and “differentially culturable” bacteria (16).

Various factors can trigger B. pseudomallei to develop VBNC, including storage in water (17), low temperature, low pH, high salinity (18), storage in the soil microcosm in the presence of high concentrations of iron (19), and treatment with disinfectants (20). Despite detailed descriptions of these factors, systematic characterization of B. pseudomallei VBNC and their resuscitation has not been attempted. We define these forms as differentially culturable bacteria (DCB), as they can only grow in certain conditions. Transition to the DC state is often accompanied by morphological changes, such as the generation of coccoid cells and the alteration of peptidoglycan structure and composition (13). Several peptidoglycan-remodeling enzymes have been previously implicated in the generation of coccoid DCB (21). Moreover, lytic transglycosylases, a family of cell-remodeling enzymes, have been shown to resuscitate DC mycobacteria (15, 22).

Here, we demonstrate that B. pseudomallei and its laboratory surrogate Burkholderia thailandensis lose the ability to produce colonies on solid agar following incubation in phosphate-buffered saline (PBS) at 4°C, but not at 21°C. We further demonstrate the generation of substantial populations of coccoid cells in these samples and confirm that they can be resuscitated in liquid media supplemented either with catalase and Tween 80 or with supernatant obtained from the logarithmic phase cultures of B. thailandensis. The application of osmoprotective media used for the propagation of cell wall-deficient bacteria (or l-forms) was partially successful and stimulated the regrowth of some starved bacteria.

In this study, we also demonstrate that a B. pseudomallei strain that has four lytic transglycosylase genes deleted cannot produce coccoid DCB and does not resuscitate in liquid media supplemented with catalase and Tween 80. Our results suggest that Ltg-mediated peptidoglycan remodeling facilitates the generation and possibly the recovery of DCB.

## RESULTS

### Low temperature induces the generation of morphologically distinct populations of *B. pseudomallei* and *B. thailandensis*.

**FIG 1:**
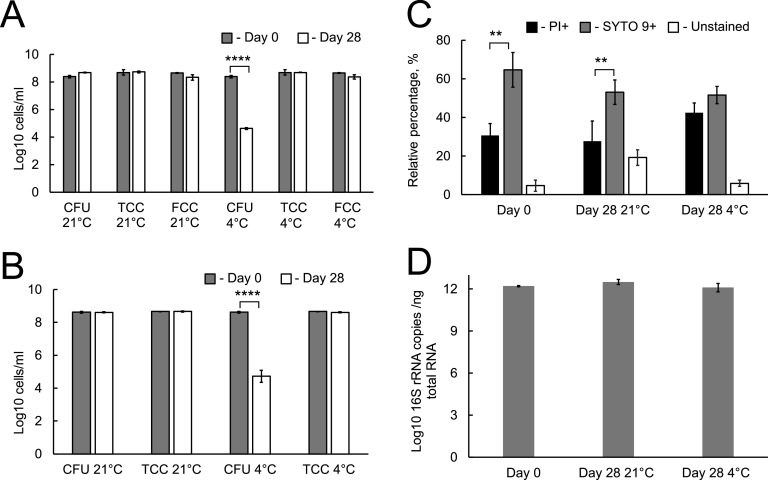
Assessment of B. thailandensis (panels A, C, D) and B. pseudomallei (panel B) survival following incubation in PBS at 21°C and 4°C for 28 days. Bacteria from late-logarithmic phase were washed in PBS and incubated at two temperatures, statically, for 28 days. (A, B) Colony-forming unit (CFU) counts were determined on LA media (also see Fig. S1); total cell counts were assessed using either a Thoma counting chamber (TCC) or flow cytometry (FCC). (C) B. thailandensis samples were stained with live/dead stains and analyzed using an Accuri Flow Cytometer. PI+, bacteria that were stained with propidium iodide; SYTO 9+, bacteria stained with SYTO 9. (D) 16S rRNA copy numbers were determined by qPCR. Data are presented as the mean ± standard error of the mean (SEM) (*n* = 3). **, *P* < 0.01; ****, *P* < 0.0001.

To investigate the effect of temperature on the survival of *Burkholderia* in nutrient-limited conditions, the bacteria were incubated in PBS for 28 days at 21°C and at 4°C (see Fig. S1 in the supplemental material). Samples of these cultures were taken over the 28-day incubation period and analyzed using growth assays, microscopy, and flow cytometry. As Fig. 1A shows, at 21°C the CFU counts for B. thailandensis did not change for the entire incubation period and remained at approximately 4 × 10^8^ CFU/mL. The total cell counts (TCC), as determined by microscopy or flow cytometry (FCC), also did not alter substantially (approximately 4.9 × 10^8^ cells/mL). In comparison, incubation at 4°C resulted in a gradual reduction in CFU count, and by day 28 there was significant reduction in CFU count compared to the starting inoculum (Fig. S1 and Fig. 1A) (*P* < 0.0001). Total cell counts measured by microscopy or flow cytometry remained unchanged (Fig. 1A). Importantly, very similar survival patterns were also observed for B. pseudomallei (Fig. 1B).

Application of live/dead staining showed that approximately 65% of B. thailandensis were stained with SYTO 9 at the start of the experiment and approximately 30% were propidium iodide (PI)-positive. Unstained bacteria represented 5% of the population. Following 28 days of incubation at either temperature, the percentage of SYTO 9-positive bacteria was reduced and they represented approximately half of the cells incubated at both temperatures (Fig. 1C), indicating that the majority of bacteria in both samples were viable. Interestingly, cultures incubated at 21°C had a substantial proportion of unstained bacteria (19%) and the sample incubated at 4°C had a higher proportion of PI-positive bacteria (approximately 28%).

We further confirmed that levels of 16S rRNA did not change significantly (*P* > 0.05) following the incubation of bacteria in PBS for 28 days at either temperature (Fig. 1D). Altogether these findings confirmed that B. thailandensis incubated at 21°C or 4°C had significant proportions of SYTO 9-positive, PI-negative cells and retained 16S rRNA, which is considered a marker of cell viability (23).

**FIG 2:**
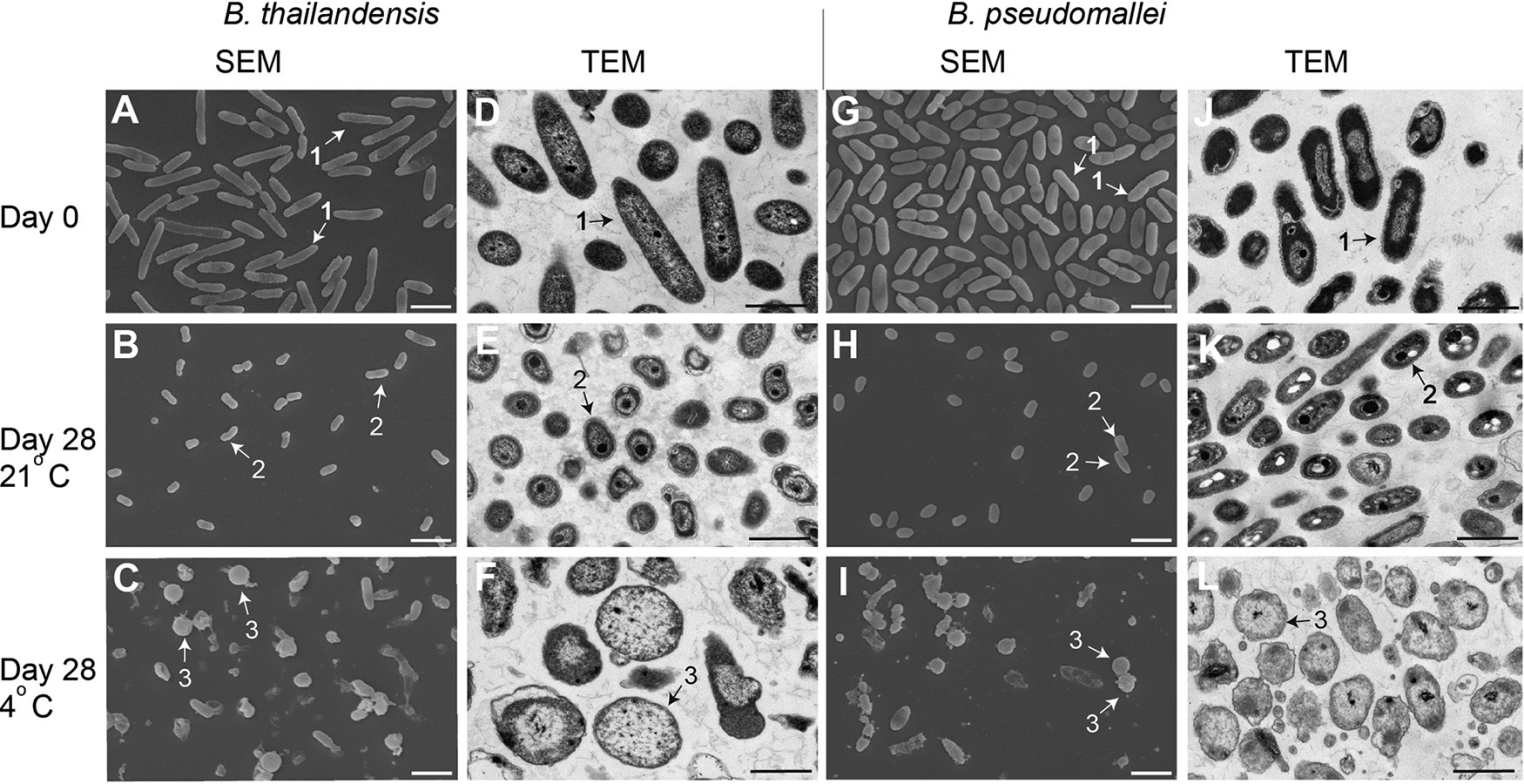
Scanning electron microscopy (SEM) and transmission electron microscopy (TEM) revealed distinct morphological features of B. thailandensis and B. pseudomallei incubated in PBS. A, D, G, J, growing bacteria; B, E, H, K, bacteria incubated at 21°C; C, F, I, L, bacteria incubated at 4°C Arrows point at specific morphological types; 1, rods; 2, short rods; 3, coccoid cells. SEM scale bars (white) are 2 µm. TEM scale bars (black) are 1 µm. Representative images shown from 4 independent experiments.

Microscopic examination of B. thailandensis revealed that the samples incubated at 21°C were dominated by short rods which were 2.4-fold shorter than bacteria growing in LB at 37°C (0.94 µm versus 2.24 µm). They had a thickened cell wall (Fig. 2, left panel) and showed no signs of substantial lysis and debris. In comparison, cultures incubated at 4°C were represented by two cell types: approximately 35% were coccoid cells with a thinner cell wall and an estimated diameter of 0.82 µm, and 65% were rods which were significantly shorter (1.35 µm) than the growing bacteria (2.21 µm; *P* < 0.0001). The samples also contained lysed cells and debris (Fig. 2, left panel). B. pseudomallei incubated under the same conditions showed very similar patterns as illustrated by Fig. 2, right panel. Thus, starvation of two *Burkholderia* species at two temperatures resulted in the generation of morphologically distinct populations.

### Coccoid cells resuscitate in liquid media supplemented with culture supernatant or catalase and Tween 80.

**FIG 3:**
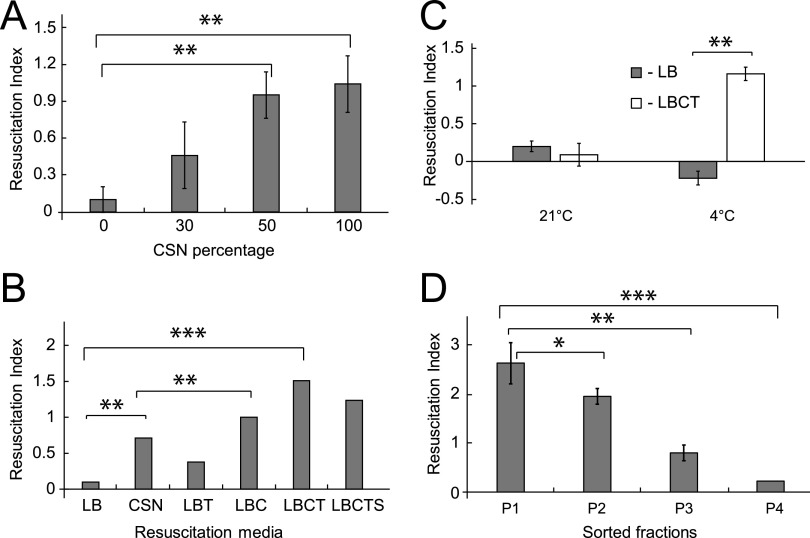
Starved *Burkholderia* resuscitate in liquid media supplemented with culture supernatant or catalase and Tween 80. (A, B, D) B. thailandensis were incubated in PBS at 4°C for 28 days and resuscitated in different liquid media. (A) Supernatants obtained from growing B. thailandensis cultures (CSN) resulted in resuscitation of starved bacteria. (B) Effect of catalase and Tween 80 on resuscitation of B. thailandensis. LB, lysogeny broth, CSN, LB+ 50% culture supernatant; LBT, LB+ 0.05% Tween 80; LBC, LB + catalase (130U/mL); LBCT, LB + catalase + Tween 80; LBCTS, LB + catalase + Tween 80 + culture supernatant. (C) Resuscitation of B. pseudomallei incubated in PBS at 4°C for 28 days. (D) Resuscitation of sorted B. thailandensis populations in LBCT medium. Resuscitation Index (RI) = Log 10 MPN-Log_10_ CFU. Data are presented as the mean ± SEM (*n* = 3). *, *P* < 0.05; **, *P* < 0.01; ***, *P* < 0.001.

The findings described above provided indirect evidence that samples incubated at 4°C contained differentially culturable cells. To confirm this observation, the resuscitation of these bacteria in liquid medium was investigated. B. thailandensis incubated at 21°C were able to grow on solid agar and in liquid media (Fig. S2A), while the cultivation of B. thailandensis incubated at 4°C in liquid LB did not result in resuscitation (Fig. 3A and B). Replacement of LB with minimal M9 based media also did not improve regrowth of DCB (data not shown).

Culture supernatants (CSN) have been previously shown to stimulate resuscitation and the growth of various bacteria (15, 24, 25). CSN were collected from various growth stages of B. thailandensis and did result in the resuscitation of bacteria (Fig. S2B). The highest resuscitation index (RI) was observed with CSN prepared from late logarithmic phase cultures (optical density [OD] ∼ 0.8) (Fig. S2B) which was either undiluted or diluted 50% in LB (Fig. 3A) (both *P* < 0.01). Undiluted CSN produced a slightly higher resuscitation index compared with 50% CSN; however, this difference was not significant.

Morphological changes suggested potential cell wall damage. The application of media developed for the cultivation of l-forms was investigated (26) and demonstrated improved regrowth of B. thailandensis samples incubated at 4°C (Fig. S2C). Catalase stimulated the growth and propagation of l-forms (27) and promoted regrowth of viable but nonculturable cells (VBNC) produced by Salmonella typhi and Vibrio vulnificus (28, 29). We therefore evaluated whether it could also improve the resuscitation of B. thailandensis DCB. Figure 3B shows that the addition of catalase significantly improved resuscitation (*P* < 0.01), while a combination of catalase and Tween 80 in LB (LBCT) media resulted in the highest resuscitation index (*P* < 0.001). Interestingly, combining catalase, Tween 80, and CSN did not further enhance resuscitation, suggesting that the active compound in CSN and catalase may have similar resuscitation-promoting mechanisms. In fact, the accumulation of catalase in CSN of B. pseudomallei and Pseudomonas aeruginosa has been previously observed (30, 31). B. pseudomallei DCB produced at 4°C were also able to resuscitate in LBCT media but not in LB (Fig. 3C) (*P* < 0.01).

As described above, the samples incubated at 4°C contained a mixture of lysed cells and rod-shaped and coccoid bacteria. Previous work has shown that the sorting of dormant bacteria improved resuscitation (32). Flow cytometry analysis identified 4 B. thailandensis populations with distinct staining patterns: P1 SYTO 9-stained bacteria with a low intensity of fluorescence (SYTO 9+LF), P2 SYTO 9-stained bacteria with a high intensity fluorescence (SYTO9+HF), P3 bacteria stained with PI and SYTO 9 (SYTO 9+PI+), and P4 bacteria stained with PI (PI+) (Fig. S3A). These populations were sorted using a FACSAria Cell Sorter and further analyzed. B. thailandensis that were able to grow on solid agar were found in the P1 to P3 populations; however, none of these samples showed significant resuscitation in LB (Fig. S3B). No live bacteria were recovered from the P4 sample. Use of the LBCT medium resulted in significant resuscitation of B. thailandensis from the P1 to P3 populations, no growth was observed in the P4 population. The highest resuscitation was achieved from the P1 population (RI = 2.63). As Fig. S3C shows, approximately 1% of the unsorted bacteria were able to resuscitate in LBCT medium, while 4.8% of bacteria could be resuscitated in LBCT from the sorted sample (P1). These observations suggest that cell sorting improved resuscitation, as previously documented for Micrococcus luteus (32), which may be due to the removal of toxic chemicals released from dead bacteria. However, the overall low percentage of resuscitating bacteria could be due to the sorting procedure itself and its effect on bacterial recovery.

Microscopic examination confirmed that the P1 sample mainly contained coccoid cells (Fig. S3C) and that the P2 sample was a combination of coccoid and rod-shaped cells (Fig. S3D). Thus, our findings confirm that SYTO 9-positive coccoid cells resuscitated in liquid media supplemented with catalase and Tween 80 or CSN.

### Lytic transglycosylases contribute to the generation and resuscitation of coccoid cells.

**FIG 4:**
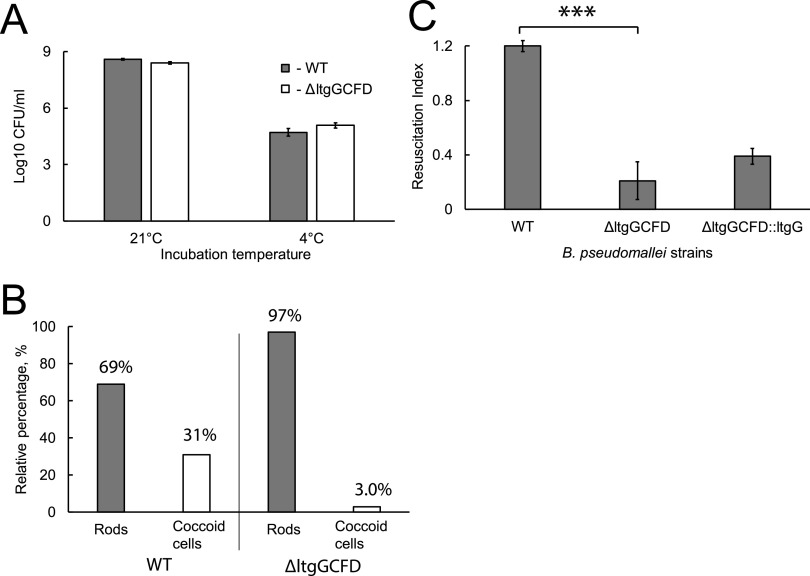
B. pseudomallei Δ*ltgGCFD* produced fewer coccoid cells and did not resuscitate in LBCT media. (A) Wild type (WT) and Δ*ltgGCFD* were incubated in PBS at 21°C and 4°C for 28 days statically. CFU counts were determined on LA. (B) Estimation of the rod and coccoid cell numbers using SEM micrographs and ImageJ. Three hundred cells were counted, and the percentages of rod and coccoid cells were determined. (C) Resuscitation was carried out with WT, Δ*ltgGCFD*, and Δ*ltgGCFD* cells complemented with *ltgG* in LBCT medium. Data are presented as the mean ± SEM (except for panel B), *n* = 3; ***, *P* < 0.001.

The coccoid cells investigated in this study demonstrated a resemblance to cells produced following treatment with certain antimicrobials and phages, suggesting potential dysregulation of the peptidoglycan-making machinery (33–35). Lytic transglycosylases (Ltgs) are a class of peptidoglycan-modifying enzymes which are believed to be activated when penicillin-binding proteins (PBP) are inhibited, contributing to penicillin-mediated lysis (36). To investigate whether these enzymes were involved in the formation of DCB, we used a B. pseudomallei quadruple *ltg*-deletion mutant (Δ*ltgGCFD)* (37) and compared its survival with that of wild-type B. pseudomallei. Both strains had similar CFU counts following incubation in PBS at either temperature (Fig. 4A). However, microscopic examination revealed some striking differences. While incubation at 21°C resulted in approximately a 4.5-fold reduction in the cell length of the mutant (Fig. S4) compared to wild type, the mutant produced a small number of coccoid cells (3% in the mutant versus 31% in the wild type) following incubation at 4°C (Fig. 4B) Resuscitation experiments showed that the mutant did not resuscitate in LBCT media and that reintroduction of the *ltgG* gene was not sufficient for complementation of this resuscitation defect (Fig. 4C). Previous studies have demonstrated that the reintroduction of *ltgG* was sufficient for complementation of division and motility defects of Δ*ltgGCFD* (37). Further experiments are required to identify the precise role of Ltgs in the generation and resuscitation of DCB.

## DISCUSSION

Bacterial dormancy and the resuscitation of dormant cells remain some of the most controversial topics of modern microbiology. It is widely believed that dormant bacteria are more tolerant to most antimicrobial agents and can persist in an infected host or in laboratory conditions for years. However, there are many different mechanisms that enable bacterial persistence, and how these align is difficult to investigate.

Differentially culturable bacteria (DCB, previously defined as VBNC) are of particular interest, as they often remain undetected and their number remains underestimated. These bacteria lose the ability to grow on solid agar and require resuscitation in liquid media. There is an ongoing discussion as to whether DCB can infect a host, as they often produce virulence factors (11, 13); however, direct demonstration of whether these cells can cause active disease is limited (38, 39). Transition to the DC state is usually accompanied by reduced metabolic activity, morphological changes, and the alteration of peptidoglycan structure and composition (13, 38).

**FIG 5:**
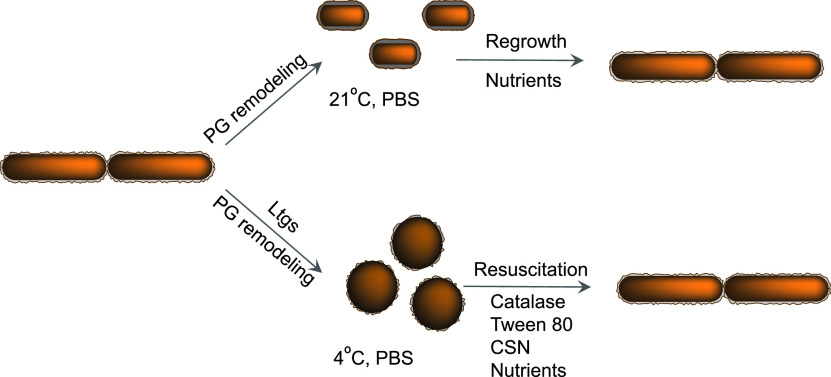
The survival mechanisms of starved *Burkholderia* are dependent on temperature. At 21°C, starved *Burkholderia* produce short rods with a thickened cell wall that rapidly regrow on solid agar or in liquid media. Following starvation at 4°C, *Burkholderia* convert into coccoid cells with thinner cell walls that can be resuscitated in liquid media supplemented with catalase and Tween 80 or culture supernatant (CSN). Both adaptation mechanisms require the remodeling of the cell wall. The generation of coccoid cells is mediated by lytic transglycosylases (Ltgs). PBS, phosphate-buffered saline.

Here, we report that *Burkholderia* utilize different starvation survival mechanisms following incubation at room temperature or 4°C. B. thailandensis *and* B. pseudomallei produce coccoid cells with specific growth requirements when starved at low temperature (Fig. 5). In comparison, incubation at 21°C leads to the generation of shorter rods with the ability to rapidly regrow in fresh media and produce colonies on solid agar. Microscopic studies revealed differences in the appearance of the cell walls of starved bacteria (Fig. 2), and future studies will aid the understanding of the specific features and mechanisms of peptidoglycan remodeling which enable *Burkholderia* to adapt to different temperatures. Our results support previous observations on the survival of *Burkholderia* under stress conditions by producing coccoid cells (18) and confirm that these coccoid cells can resuscitate in liquid media containing either culture supernatant or catalase and Tween 80 (Fig. 3). Catalase was previously shown to stimulate the resuscitation of VBNC produced by various Gram-negative bacteria (23, 29) and to promote the replication of l-form bacteria (40). In both systems, bacteria likely experienced metabolic imbalance and increased generation of reactive oxygen species (ROS), the damaging effect of which was reduced by catalase. The reactivation of coccoid forms of B. thailandensis and B. pseudomallei in nutrient-rich medium is likely accompanied by increased metabolism and generation of ROS. The addition of Tween 80 could help to disperse stored bacteria, provide an alternative nonglycolytic nutrient source, or stimulate the production of lipase (41) for removal of damaged lipids. The resuscitation effect of culture supernatant is likely caused by catalase; however, we cannot exclude that other resuscitation moieties are released by actively growing bacteria. Resuscitation-promoting factor proteins, lytic transglycosylases in Actinobacteria, and muropeptides have been previously shown to resuscitate dormant mycobacteria (15, 22, 42). However, Gram-negative bacteria have highly efficient systems for the recycling of muropeptides (as reviewed in 43), and therefore they are unlikely to be present in the CSN of *Burkholderia*; lytic transglycosylases are secreted into the periplasm and not released in CSN (36).

Application of flow cytometry enabled the separation of coccoid cells, and our data suggest that the resuscitating population is represented by coccoid cells with low SYTO 9 fluorescence (Fig. 3D), which may be indicative of a reduction of nucleic acids and metabolic activity. Our data support recently published results obtained with Vibrio parahaemolyticus showing that bacteria starved in cold conditions produced distinct populations with differential resuscitation patterns, which correlated with unique proteomic profiles (44). We did not investigate the proteomic or transcriptomic profiles of starved bacteria; however, our data indicate that the 16S rRNA levels remained constant during incubation.

Our results suggest that coccoid *Burkholderia* combine characteristics that resemble l-forms (coccoid shape and improved growth on osmoprotective media or in media containing catalase) and features of dormant bacteria (reduced staining with SYTO 9 and the requirement for resuscitation). l-forms are bacteria which temporarily lose their cell wall, and it is currently believed that their active metabolism enables the increased synthesis of membrane material and survival. (26). However, the coccoid forms described in this study are distinct from l-forms; they contain a cell wall and can survive in non-osmoprotective media.

The precise mechanism for the generation of coccoid cells is unknown, but the observed change of shape suggests an alteration to the cell wall and the involvement of peptidoglycan modifying enzymes. It has been previously demonstrated that the composition and structure of peptidoglycan from VBNC is different (45, 46). For example, the peptidoglycan of E. coli VBNC induced by incubation at 4°C was characterized by an increased percentage of peptidoglycan cross-linking and an abundance of uncommon m-DAP-m-DAP cross-links due to an upregulation of L,d-transpeptidase (45). Additionally, these VBNC displayed a higher autocatalytic capability, suggesting the induction of peptidoglycan-cleaving enzymes and the noticeable absence of certain penicillin-binding proteins. Recent transcriptomics analysis of Pseudomonas syringae pv. *syringae* VBNC confirmed the upregulation of several genes involved in peptidoglycan biosynthesis and provided indirect evidence for the critical contribution of peptidoglycan biosynthesis and remodeling in the transition to VBNC (47). It was earlier proposed that cell wall-modifying and hydrolyzing enzymes may play a critical role in the generation of coccoid VBNC (21). Indeed, deletion of *amiA1* encoding *N*-acetyl-muramoyl-l-alanine amidase abrogated the formation of coccoid cells in Helicobacter pylori (48) and Campylobacter jejuni (49). Mutations in L,d-carboxypeptidase Pgp1, RodA, and Pbp2 were also reported to interfere with the generation of coccoid cells (reviewed in reference 21).

Here, we demonstrate that another class of peptidoglycan-modifying enzymes, lytic transglycosylases (Ltgs), contribute to the generation of DCB in B. pseudomallei (Fig. 4). Ltgs cleave the bonds between *N*-acetyl-muramic acid and *N*-acetylglucosamine and have multiple roles in bacterial physiology (36). B. pseudomallei produces at least 8 Ltgs and one of them, LtgG, has been implicated in the control of cell division, virulence, and motility (37). A quadruple Δ*ltgGCFD* mutant had a clear chaining phenotype, indicative of a growth division defect, and impairments of motility and antimicrobial susceptibility (37). We show that this mutant also had a limited capacity to produce coccoid DCB, and no resuscitation of the mutant was observed in LBCT. Reintroduction of the *ltgG* gene did not complement the mutant phenotype, suggesting that other Ltgs might be necessary for the generation and resuscitation of coccoid DCB. Complementation of multiple-deletion mutants is technically challenging, and the investigation of single-deletion mutants may reveal Ltgs important for DCB formation. We hypothesize that, at a low temperature, the peptidoglycan machinery of B. thailandensis, B. pseudomallei, and other Gram-negative bacteria is dysregulated due to the inactivation or downregulation of penicillin-binding proteins, and that the ongoing activity of cell-cleaving enzymes (in particular, Ltg) results in a change of peptidoglycan and the formation of coccoid cells. However, additional experiments are required to address our hypothesis.

In conclusion, we identified temperature as a factor that triggers the transition of *Burkholderia* into a differentially culturable state resulting in the generation of coccoid cells. A well-known seasonal charter of melioidosis has been linked to temperature (50) and our results reveal an additional temperature-dependent mechanism that may contribute to B. pseudomallei survival in the environment and influence infections in humans. The generation of DCB in B. pseudomallei can also explain the endemic nature of melioidosis, which is prevalent in tropical countries and rarely diagnosed in countries with a cold climate. However, global warming, resulting in extreme weather events and sudden changes of temperature, has a dramatic effect on microorganisms (51) and brings a new dimension to the VBNC and DCB phenomena. DCB may become a major adaptation strategy for certain pathogens, allowing their survival in extreme temperatures, while existing in multimicrobial communities may promote resuscitation; for example, by the release of catalase and other resuscitation-promoting moieties.

## MATERIALS AND METHODS

### Bacterial strains and media.

B. thailandensis E555 and B. pseudomallei K96243 were grown in Lysogeny broth (LB, Melford Biolaboratories Ltd.). Ashdown’s medium was used for confirmation of *Burkholderia* and Lysogeny agar (Melford Biolaboratories Ltd.) was used for determination of CFU counts. B. pseudomallei K96243 Δ*ltgGCFD*, Δ*ltgGCFD*::pM030-*ltgG* mutants (37) were handled as previously described. All bacterial cultures were cultivated at 37°C with shaking at 200 rpm until required growth phase.

### Starvation experiments.

*Burkholderia* cultures from the late logarithmic phase (OD_600_ of 1.0 to 1.5) were washed in PBS (Fisher Scientific) three times and resuspended in PBS to a final OD of 0.9, and 15 mL of this culture was aliquoted into 50-mL Falcon tubes. The tubes were sealed with Parafilm and incubated statically at 21°C and 4°C.

### Growth and resuscitation assays.

For CFU counting, bacteria were serially diluted in PBS; four 10-µL drops of each dilution were spotted on agar and, when dry, the plates were incubated at 37°C for 24 h before colonies were enumerated.

For Most Probable Number (MPN) counts, 50-µL volumes were added to four wells of a 48-well plate containing 450 µL of media (22), and serially diluted using 50-µL volumes for up to 10^−9^ dilution. Plates were sealed and incubated statically at 37°C. Positive wells were scored and MPN counts were determined using the MPN calculator program (52).

For resuscitation assays, several media were tested: (i) LB, (ii) LB containing 30% and 50% CSN, (iii) undiluted CSN, (iv) LB containing catalase 130 U/mL and 0.05% (vol/vol) Tween 80, and (v) LB containing 0.3 M sucrose, 20 mM maleic acid, and 20 mM MgCl_2_.

CSN was prepared from logarithmic phase cultures (OD ∼ 0.8 to 1.0) grown in LB. Cultures were centrifuged at 4000 × *g* for 15 min and sterilized by passing through a 0.22-µm filter. CSN were used for experiments on the same day. The resuscitation index was calculated using the following formula:

Resuscitation Index(RI) = Log10(MPN/mL)−Log10(CFU/mL).

### Microscopy.

A Ti-Eclipse phase microscope (Nikon) and a 12/10 bit, high-speed, Peltier-cooled CCD camera (FDI, Photonic Science) were used for visualization and imaging of unstained bacteria. A Thoma-type bacterial cell-counting chamber (Hawksley Z30000 Helber) was used for the estimation of total bacterial counts. Images were analyzed using the NIS-Elements imaging software (Nikon) and Image J (53).

For electron microscopy, pellets obtained from 15 ml cultures were fixed in 2.5% (vol/vol) glutaraldehyde in PBS for 24 h.

For SEM, cells were applied to glass coverslips and postfixed in 0.5% (wt/vol) aqueous osmium before dehydration through a graded series of ethanol and hexamethyldisilazane. Coverslips were mounted onto aluminum stubs, sputter-coated with gold/palladium (Quorum Q150T ES, 90 s, 20 mA), and visualized on a Hitachi S300H SEM with an accelerating voltage of 10 kV. Measurement of cell length was performed using Fiji version 2.0.0-rc69/1.52p software. Three hundred cells were counted for each sample. Fiji 2.0.0-rc69/1.52p with BioVoxel plugin was applied to distinguish rod and coccoid cells.

For transmission electron microscopy (TEM), cells were postfixed in 1% (wt/vol) aqueous osmium, dehydrated through ethanol, and embedded in Spurr’s resin. The 70-nm resin sections were cut with an ultramicrotome (Reichert Ultracut E), collected onto copper mesh grids, and stained with 2% aqueous uranyl acetate followed by lead citrate. Samples were visualized on a JEOL JEM-1400 TEM with an accelerating voltage of 100 kV. Digital images were collected with a Megaview III digital camera with iTEM software (EMSIS, Germany).

### Flow cytometry and sorting.

A BD FACSAria Flow cytometer and sorter (BD Biosciences) was used to analyze cell populations in starved B. thailandensis samples. Bacteria were stained with SYTO 9 and the Propidium Iodide Live/Dead staining kit (Thermo Fisher Scientific) as described previously (54). A total of 20,000 events were measured per sample. Excitation of SYTO 9 occurred at 488 nm while emission was 530/540 nm. Excitation of propidium iodide occurred at 561 nm while emission was 610/620 nm. The instrument was calibrated using beads and protocols provided by the manufacturer.

### RNA isolation.

RNA was isolated from starved bacteria using the GTC TRIzol protocol (55). RT-qPCR was performed in a Corbett Rotor Gene 6000 real-time PCR machine using RNA superscript and a SYBR Green mix (Thermo Fisher Scientific). 16S rRNA copy numbers were normalized per ng of total RNA. The following B. thailandensis specific primers were purchased from Sigma-Aldrich and used for the assessment of 16S rRNA in starved bacteria: 16S rRNAF 5′-GACACGGCCCAGACTCCTAC-3′ and 16S rRNAR 5′-CCGGTACCGTCATCCACTCC-3′.

## References

[1] Coenye T, Vandamme P. 2003. Diversity and significance of *Burkholderia* species occupying diverse ecological niches. Environ Microbiol 5:719–729. doi:10.1046/j.1462-2920.2003.00471.x. 12919407

[2] Limmathurotsakul D, Golding N, Dance DA, Messina JP, Pigott DM, Moyes CL, Rolim DB, Bertherat E, Day NP, Peacock SJ, Hay SI. 2016. Predicted global distribution of *Burkholderia pseudomallei* and burden of melioidosis. Nat Microbiol 1:15008. doi:10.1038/nmicrobiol.2015.8. 27571754

[3] Domthong P, Chaisuksant S, Sawanyawisuth K. 2016. What clinical factors are associated with mortality in septicemic melioidosis? A report from an endemic area. J Infect Dev Ctries 10:404–409. doi:10.3855/jidc.6455. 27131004

[4] Johnson AB, Ali N. 1990. Reactivation of latent melioidosis. Postgrad Med J 66:732–733. doi:10.1136/pgmj.66.779.732. 2235805 PMC2426895

[5] Yip TW, Hewagama S, Mayo M, Price EP, Sarovich DS, Bastian I, Baird RW, Spratt BG, Currie BJ. 2015. Endemic melioidosis in residents of desert region after atypically intense rainfall in central Australia. Emerg Infect Dis 21:1038–1040. doi:10.3201/eid2106.141908. 25988301 PMC4451904

[6] Webb JR, Buller N, Rachlin A, Golledge C, Sarovich DS, Price EP, Mayo M, Currie BJ. 2020. A persisting nontropical focus of *Burkholderia pseudomallei* with limited genome evolution over five decades. mSystems 5. doi:10.1128/mSystems.00726-20. PMC7657595 33172968

[7] Cossaboom CM, Marinova-Petkova A, Strysko J, Rodriguez G, Maness T, Ocampo J, Gee JE, Elrod MG, Gulvik CA, Liu L, Bower WA, Hoffmaster AR, Blaney DD, Salzer JS, Yoder JS, Mattioli MC, Sidwa TJ, Ringsdorf L, Morrow G, Ledezma E, Kieffer A. 2020. Melioidosis in a resident of Texas with no recent travel history, United States. Emerg Infect Dis 26:1295–1299. doi:10.3201/eid2606.190975. 32442394 PMC7258475

[8] Hantrakun V, Rongkard P, Oyuchua M, Amornchai P, Lim C, Wuthiekanun V, Day NP, Peacock SJ, Limmathurotsakul D. 2016. Soil nutrient depletion is associated with the presence of *Burkholderia pseudomallei*. Appl Environ Microbiol 82:7086–7092. doi:10.1128/AEM.02538-16. 27694236 PMC5118919

[9] Nierman WC, Yu Y, Losada L. 2015. The *in vitro* antibiotic tolerant persister population in *Burkholderia pseudomallei* is altered by environmental factors. Front Microbiol 6:1338. doi:10.3389/fmicb.2015.01338. 26696964 PMC4678198

[10] Inglis TJ, Sagripanti JL. 2006. Environmental factors that affect the survival and persistence of *Burkholderia pseudomallei*. Appl Environ Microbiol 72:6865–6875. doi:10.1128/AEM.01036-06. 16980433 PMC1636198

[11] Oliver JD. 2010. Recent findings on the viable but nonculturable state in pathogenic bacteria. FEMS Microbiol Rev 34:415–425. doi:10.1111/j.1574-6976.2009.00200.x. 20059548

[12] Kell DB, Kaprelyants AS, Weichart D, Harwood CR, Barer MR. 1998. Viability and activity in readily culturable bacteria: a review and discussion of the practical issues. Antonie Van Leeuwenhoek 73:169–187. doi:10.1023/a:1000664013047. 9717575

[13] Ramamurthy T, Ghosh A, Pazhani GP, Shinoda S. 2014. Current perspectives on viable but non-culturable (VBNC) pathogenic bacteria. Front Public Health 2:103. doi:10.3389/fpubh.2014.00103. 25133139 PMC4116801

[14] Mukamolova GV, Kaprelyants AS, Kell DB, Young M. 2003. Adoption of the transiently non-culturable state: a bacterial survival strategy? Adv Microb Physiol 47:65–129. doi:10.1016/s0065-2911(03)47002-1. 14560663

[15] Shleeva MO, Bagramyan K, Telkov MV, Mukamolova GV, Young M, Kell DB, Kaprelyants AS. 2002. Formation and resuscitation of “non-culturable” cells of *Rhodococcus rhodochrous* and *Mycobacterium tuberculosis* in prolonged stationary phase. Microbiology (Reading) 148:1581–1591. doi:10.1099/00221287-148-5-1581. 11988533

[16] Chengalroyen MD, Beukes GM, Gordhan BG, Streicher EM, Churchyard G, Hafner R, Warren R, Otwombe K, Martinson N, Kana BD. 2016. Detection and quantification of differentially culturable tubercle bacteria in sputum from patients with tuberculosis. Am J Respir Crit Care Med 194:1532–1540. doi:10.1164/rccm.201604-0769OC. 27387272 PMC5215032

[17] Gilbert SE, Rose LJ. 2012. Survival and persistence of nonspore-forming biothreat agents in water. Lett Appl Microbiol 55:189–194. doi:10.1111/j.1472-765X.2012.03277.x. 22725260

[18] Robertson J, Levy A, Sagripanti JL, Inglis TJ. 2010. The survival of *Burkholderia pseudomallei* in liquid media. Am J Trop Med Hyg 82:88–94. doi:10.4269/ajtmh.2010.09-0226. 20065001 PMC2803515

[19] Taweechaisupapong S, Kamjumphol W, Chareonsudjai S, Chareonsudjai P. 2015. Morphological alteration and survival of *Burkholderia pseudomallei* in soil microcosms. Am J Trop Med Hyg 93:1058–1065. doi:10.4269/ajtmh.15-0177. 26324731 PMC4703280

[20] O'Connell HA, Rose LJ, Shams A, Bradley M, Arduino MJ, Rice EW. 2009. Variability of *Burkholderia pseudomallei* strain sensitivities to chlorine disinfection. Appl Environ Microbiol 75:5405–5409. doi:10.1128/AEM.00062-09. 19542324 PMC2725453

[21] Ikeda N, Karlyshev AV. 2012. Putative mechanisms and biological role of coccoid form formation in *Campylobacter jejuni*. Eur J Microbiol Immunol (Bp) 2:41–49. doi:10.1556/EuJMI.2.2012.1.7. 24611120 PMC3933989

[22] Mukamolova GV, Turapov O, Malkin J, Woltmann G, Barer MR. 2010. Resuscitation-promoting factors reveal an occult population of tubercle bacilli in sputum. Am J Respir Crit Care Med 181:174–180. doi:10.1164/rccm.200905-0661OC. 19875686 PMC2809243

[23] Zhang S, Guo L, Yang K, Zhang Y, Ye C, Chen S, Yu X, Huang WE, Cui L. 2018. Induction of *Escherichia coli* into a VBNC state by continuous-flow UVC and subsequent changes in metabolic activity at the single-cell level. Front Microbiol 9:2243. doi:10.3389/fmicb.2018.02243. 30319570 PMC6167417

[24] Weichart DH, Kell DB. 2001. Characterization of an autostimulatory substance produced by *Escherichia coli*. Microbiology (Reading) 147:1875–1885. doi:10.1099/00221287-147-7-1875. 11429464

[25] Pascoe B, Dams L, Wilkinson TS, Harris LG, Bodger O, Mack D, Davies AP. 2014. Dormant cells of *Staphylococcus* aureus are resuscitated by spent culture supernatant. PLoS One 9:e85998. doi:10.1371/journal.pone.0085998. 24523858 PMC3921112

[26] Kawai Y, Mercier R, Mickiewicz K, Serafini A, Sorio de Carvalho LP, Errington J. 2019. Crucial role for central carbon metabolism in the bacterial L-form switch and killing by beta-lactam antibiotics. Nat Microbiol 4:1716–1726. doi:10.1038/s41564-019-0497-3. 31285586 PMC6755032

[27] Kawai Y, Mercier R, Wu LJ, Dominguez-Cuevas P, Oshima T, Errington J. 2015. Cell growth of wall-free L-form bacteria is limited by oxidative damage. Curr Biol 25:1613–1618. doi:10.1016/j.cub.2015.04.031. 26051891 PMC4510147

[28] Zeng B, Zhao G, Cao X, Yang Z, Wang C, Hou L. 2013. Formation and resuscitation of viable but nonculturable *Salmonella typhi*. Biomed Res Int 2013:907170. doi:10.1155/2013/907170. 23509799 PMC3591152

[29] Bogosian G, Aardema ND, Bourneuf EV, Morris PJ, O'Neil JP. 2000. Recovery of hydrogen peroxide-sensitive culturable cells of *Vibrio vulnificus* gives the appearance of resuscitation from a viable but nonculturable state. J Bacteriol 182:5070–5075. doi:10.1128/JB.182.18.5070-5075.2000. 10960089 PMC94653

[30] Vellasamy KM, Vasu C, Puthucheary SD, Vadivelu J. 2009. Comparative analysis of extracellular enzymes and virulence exhibited by *Burkholderia pseudomallei* from different sources. Microb Pathog 47:111–117. doi:10.1016/j.micpath.2009.06.003. 19524661

[31] Hassett DJ, Alsabbagh E, Parvatiyar K, Howell ML, Wilmott RW, Ochsner UA. 2000. A protease-resistant catalase, KatA, released upon cell lysis during stationary phase is essential for aerobic survival of a Pseudomonas aeruginosa oxyR mutant at low cell densities. J Bacteriol 182:4557–4563. doi:10.1128/JB.182.16.4557-4563.2000. 10913089 PMC94627

[32] Kaprelyants AS, Mukamolova GV, Davey HM, Kell DB. 1996. Quantitative analysis of the physiological heterogeneity within starved cultures of *Micrococcus luteus* by flow cytometry and cell sorting. Appl Environ Microbiol 62:1311–1316. doi:10.1128/aem.62.4.1311-1316.1996. 16535295 PMC1388833

[33] Lennings J, Makhlouf M, Olejnik P, Mayer C, Brotz-Oesterhelt H, Schwarz S. 2019. Environmental and cellular factors affecting the localization of T6SS proteins in *Burkholderia thailandensis*. Int J Med Microbiol 309:151335. doi:10.1016/j.ijmm.2019.151335. 31378704

[34] van Teeseling MCF, de Pedro MA, Cava F. 2017. Determinants of bacterial morphology: from fundamentals to possibilities for antimicrobial targeting. Front Microbiol 8:1264. doi:10.3389/fmicb.2017.01264. 28740487 PMC5502672

[35] Turnbull L, Toyofuku M, Hynen AL, Kurosawa M, Pessi G, Petty NK, Osvath SR, Carcamo-Oyarce G, Gloag ES, Shimoni R, Omasits U, Ito S, Yap X, Monahan LG, Cavaliere R, Ahrens CH, Charles IG, Nomura N, Eberl L, Whitchurch CB. 2016. Explosive cell lysis as a mechanism for the biogenesis of bacterial membrane vesicles and biofilms. Nat Commun 7:11220. doi:10.1038/ncomms11220. 27075392 PMC4834629

[36] Dik DA, Marous DR, Fisher JF, Mobashery S. 2017. Lytic transglycosylases: concinnity in concision of the bacterial cell wall. Crit Rev Biochem Mol Biol 52:503–542. doi:10.1080/10409238.2017.1337705. 28644060 PMC6102726

[37] Jenkins CH, Wallis R, Allcock N, Barnes KB, Richards MI, Auty JM, Galyov EE, Harding SV, Mukamolova GV. 2019. The lytic transglycosylase, LtgG, controls cell morphology and virulence in *Burkholderia pseudomallei*. Sci Rep 9:11060. doi:10.1038/s41598-019-47483-z. 31363151 PMC6667503

[38] Zhao X, Zhong J, Wei C, Lin CW, Ding T. 2017. Current perspectives on viable but non-culturable state in foodborne pathogens. Front Microbiol 8:580. doi:10.3389/fmicb.2017.00580. 28421064 PMC5378802

[39] Kell D, Potgieter M, Pretorius E. 2015. Individuality, phenotypic differentiation, dormancy and 'persistence' in culturable bacterial systems: commonalities shared by environmental, laboratory, and clinical microbiology. F1000Res 4:179. doi:10.12688/f1000research.6709.2. 26629334 PMC4642849

[40] Errington J, Mickiewicz K, Kawai Y, Wu LJ. 2016. L-form bacteria, chronic diseases and the origins of life. Philos Trans R Soc B 371:20150494. doi:10.1098/rstb.2015.0494. PMC5052740 27672147

[41] Boekema BK, Beselin A, Breuer M, Hauer B, Koster M, Rosenau F, Jaeger KE, Tommassen J. 2007. Hexadecane and Tween 80 stimulate lipase production in *Burkholderia glumae* by different mechanisms. Appl Environ Microbiol 73:3838–3844. doi:10.1128/AEM.00097-07. 17468265 PMC1932709

[42] Nikitushkin VD, Demina GR, Kaprelyants AS. 2016. Rpf proteins are the factors of reactivation of the dormant form of Actinobacteria. Biochemistry Moscow 81:1719–1734. doi:10.1134/S0006297916130095. 28260493

[43] Irazoki O, Hernandez SB, Cava F. 2019. Peptidoglycan muropeptides: release, perception, and functions as signaling molecules. Front Microbiol 10:500. doi:10.3389/fmicb.2019.00500. 30984120 PMC6448482

[44] Wagley S, Morcrette H, Kovacs-Simon A, Yang ZR, Power A, Tennant RK, Love J, Murray N, Titball RW, Butler CS. 2021. Bacterial dormancy: a subpopulation of viable but non-culturable cells demonstrates better fitness for revival. PLoS Pathog 17:e1009194. doi:10.1371/journal.ppat.1009194. 33439894 PMC7837498

[45] Signoretto C, Lleo M, Canepari P. 2002. Modification of the peptidoglycan of *Escherichia coli* in the viable but nonculturable state. Curr Microbiol 44:125–131. doi:10.1007/s00284-001-0062-0. 11815857

[46] Signoretto C, Lleo M, Tafi MC, Canepari P. 2000. Cell wall chemical composition of *Enterococcus faecalis* in the viable but nonculturable state. Appl Environ Microbiol 66:1953–1959. doi:10.1128/AEM.66.5.1953-1959.2000. 10788366 PMC101439

[47] Postnikova OA, Shao J, Mock NM, Baker CJ, Nemchinov LG. 2015. Gene expression profiling in viable but nonculturable (VBNC) cells of *Pseudomonas syringae* pv. *syringae*. Front Microbiol 6:1419. doi:10.3389/fmicb.2015.01419. 26733964 PMC4683178

[48] Chaput C, Ecobichon C, Cayet N, Girardin SE, Werts C, Guadagnini S, Prevost MC, Mengin-Lecreulx D, Labigne A, Boneca IG. 2006. Role of AmiA in the morphological transition of *Helicobacter pylori* and in immune escape. PLoS Pathog 2:e97. doi:10.1371/journal.ppat.0020097. 17002496 PMC1574363

[49] Frirdich E, Biboy J, Pryjma M, Lee J, Huynh S, Parker CT, Girardin SE, Vollmer W, Gaynor EC. 2019. The *Campylobacter jejuni* helical to coccoid transition Involves changes to peptidoglycan and the ability to elicit an immune response. Mol Microbiol 112:280–301. doi:10.1111/mmi.14269. 31070821 PMC6767375

[50] Egilmez HI, Morozov AY, Galyov EE. 2021. Modelling the spatiotemporal complexity of interactions between pathogenic bacteria and a phage with a temperature-dependent life cycle switch. Sci Rep 11:4382. doi:10.1038/s41598-021-83773-1. 33623124 PMC7902855

[51] Cavicchioli R, Ripple WJ, Timmis KN, Azam F, Bakken LR, Baylis M, Behrenfeld MJ, Boetius A, Boyd PW, Classen AT, Crowther TW, Danovaro R, Foreman CM, Huisman J, Hutchins DA, Jansson JK, Karl DM, Koskella B, Mark Welch DB, Martiny JBH, Moran MA, Orphan VJ, Reay DS, Remais JV, Rich VI, Singh BK, Stein LY, Stewart FJ, Sullivan MB, van Oppen MJH, Weaver SC, Webb EA, Webster NS. 2019. Scientists’ warning to humanity: microorganisms and climate change. Nat Rev Microbiol 17:569–586. doi:10.1038/s41579-019-0222-5. 31213707 PMC7136171

[52] Jarvis B, Wilrich C, Wilrich PT. 2010. Reconsideration of the derivation of Most Probable Numbers, their standard deviations, confidence bounds and rarity values. J Appl Microbiol 109:1660–1667. doi:10.1111/j.1365-2672.2010.04792.x. 20602657

[53] Schneider C, Rasband W, Eliceiri K. 2012. NIH Image to ImageJ: 25 years of image analysis. Nat Methods 9:671–675. doi:10.1038/nmeth.2089. 22930834 PMC5554542

[54] Khan MM, Pyle BH, Camper AK. 2010. Specific and rapid enumeration of viable but nonculturable and viable-culturable gram-negative bacteria by using flow cytometry. Appl Environ Microbiol 76:5088–5096. doi:10.1128/AEM.02932-09. 20543046 PMC2916471

[55] Waddell SJ, Butcher PD. 2010. Use of DNA arrays to study transcriptional responses to antimycobacterial compounds. *In* Antibiotic Resistance Protocols, Gillespie SH, McHugh TD (eds). Methods Mol Biol 642:75–91. Humana Press, Clifton NJ. 10.1007/978-1-60327-279-7_6 20401587

