# Supplementary material for: Generation of Distinct Differentially Culturable Forms of Burkholderia following Starvation at Low Temperature
Source: Microbiol Spectr. 2022 Jan 5;10(1):e02110-21. doi: 10.1128/spectrum.02110-21 (PMC8729786; doi:10.1128/spectrum.02110-21)
Supplement: SUPPLEMENTAL FILE 1 — Supplemental material. Download SPECTRUM02110-21_Supp_1_seq1.pdf, PDF file, 1.2 MB [file spectrum02110-21_supp_1_seq1.pdf]

Generation of distinct differentially culturable forms of *Burkholderia* during starvation at low temperature

Joss M. Auty[1], Christopher H. Jenkins[2], Jennifer Hincks[3], Anna A. Straatman-Iwanowska[4], Natalie Allcock[4], Obolbek Turapov[1], Edouard E. Galyov[5], Sarah V. Harding[1,2], Galina V. Mukamolova[1]*

[1]Department of Respiratory Sciences, University of Leicester, Leicester, UK; [2]Defence Science and Technology Laboratory, Chemical, Biological and Radiological Division, Porton Down, Salisbury, Wiltshire, UK; [3]FACS Facility Core Biotechnology Services, University of Leicester, Leicester, UK;[4]Electron Microscopy Facility, Core Biotechnology Services, University of Leicester, Leicester, UK; [5]Department of Genetics and Genome Biology, University of Leicester, Leicester, UK

Supplemental Material

4 Figures

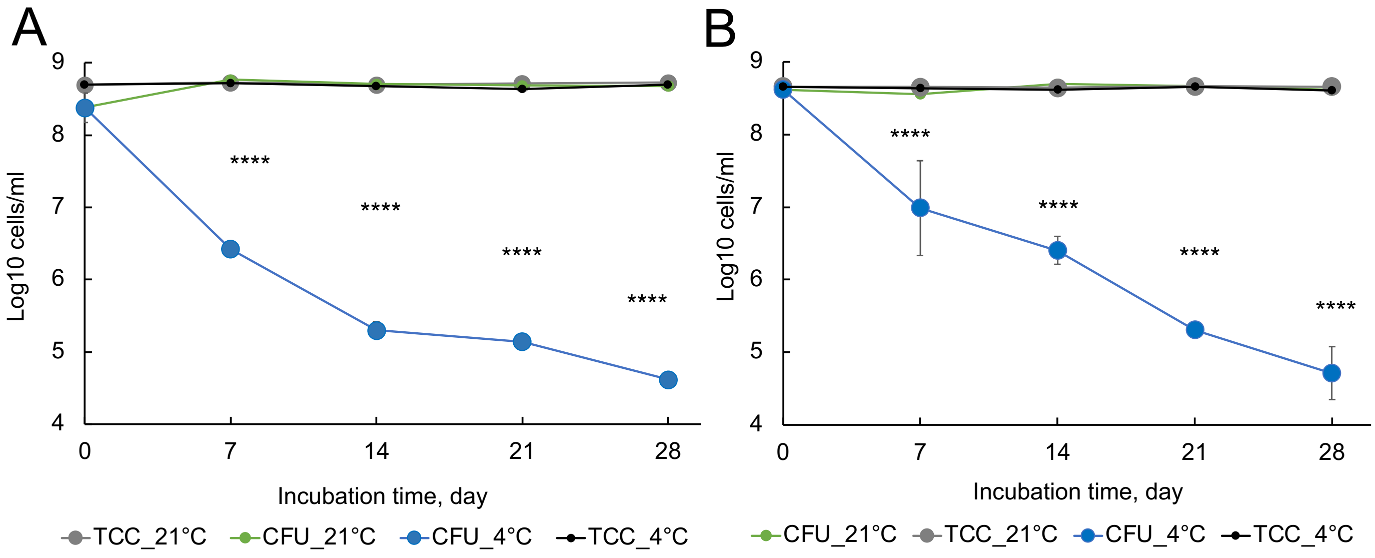

Figure S1: **Survival of *B. thailandensis* (A) or *B. pseudomallei* (B) in PBS.** Bacteria from late-logarithmic phase were washed in PBS and incubated at 21°C or 4°C statically for 28 days. Samples were taken weekly and the CFU and Total cell count (TCC) determined. Three independent experiments were performed with three replicates in each experiment. **** ($p<0.0001$).

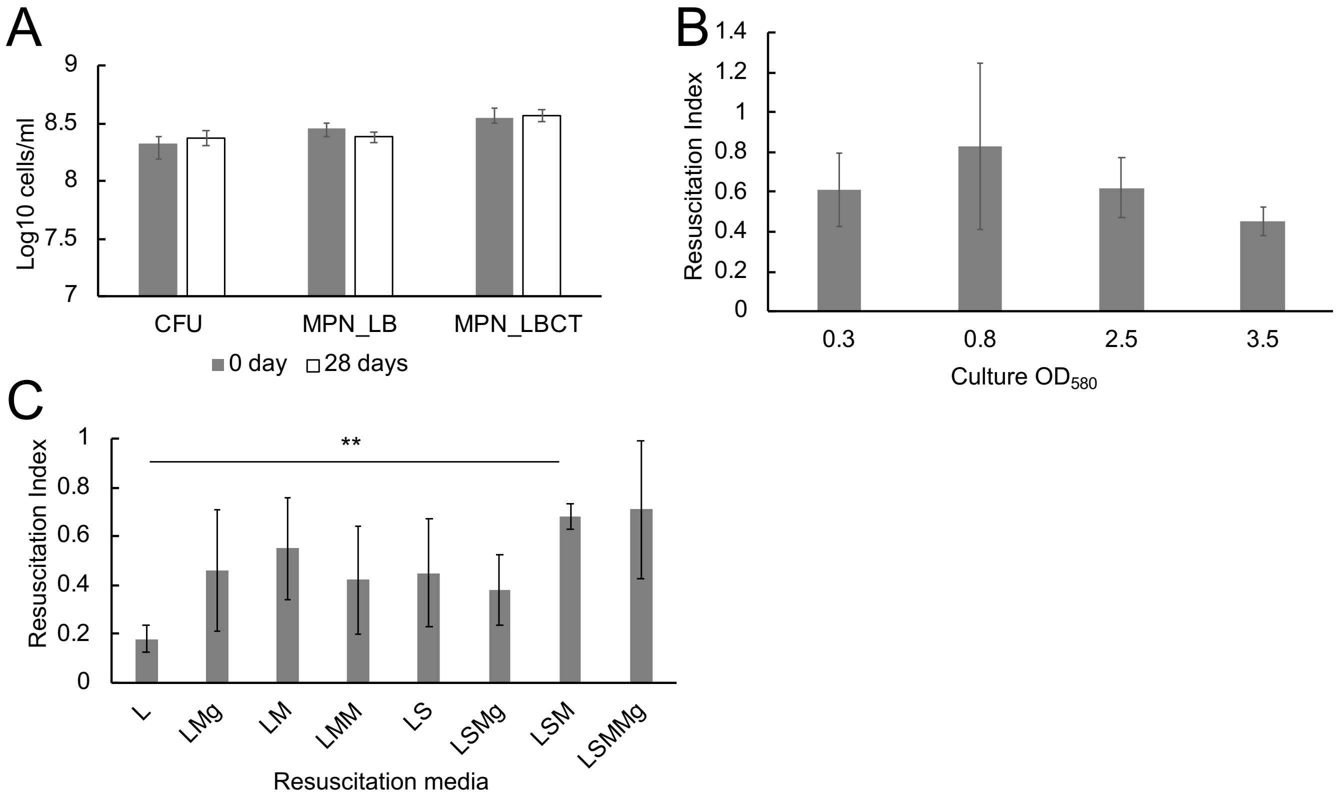

Figure S2. Investigation of the resuscitation of *B. thailandensis* in liquid media. (A) Effect of media on the resuscitation of bacteria incubated at 21°C. CFU- colony-forming unit; MPN_LB – Most Probable Number in Lysogeny broth; MPN_LBCT – Most Probable Number in Lysogeny broth containing catalase (130U/ml) and 0.05 % Tween 80. (B) Effect of growth phase on the resuscitation index of culture filtrate. (C) Media for the cultivation of L-forms improved the growth of starved bacteria. L – LB; LMg LB with 20 mM $MgCl_2$; LM – LB with 20 mM maleic acid; LMM LB with 20 mM $MgCl_2$ and 20 mM maleic acid; LS – LB with 0.3 M sucrose; LSMg – LB with 0.3 M sucrose and 20 mM $MgCl_2$; LSM – LB with 0.3 M sucrose and 20 mM maleic acid; LSMMg - LB with 0.3 M sucrose, 20 mM maleic acid and 20 mM $MgCl_2$. Data are presented as the mean ± SEM (n=3).** ($p<0.01$).

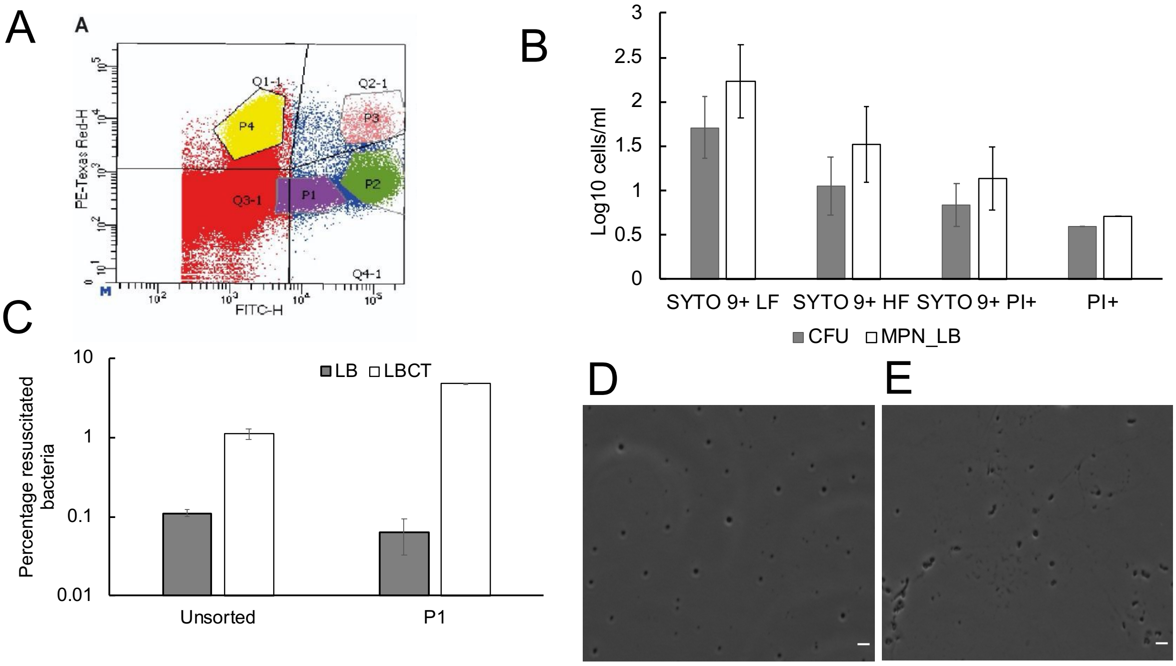

Figure S3. Sorting of starved *B. thailandensis*. (A) A schematic of four sorted populations (P1 to P4). (B) CFU and MPN counts in LB. (P1) SYTO 9+LF – cells stained with SYTO 9 with low fluorescence; (P2) SYTO 9+ HF- cells stained with SYTO 9 with high fluorescence; (P3) SYTO 9+ PI+ - cell stained with SYTO 9 and Propidium Iodide; (P4) PI+ - cells stained with Propidium Iodide. PI+ sample produced no growth on agar or in liquid media; bars shows the limits of detection. (C) Percentages of resuscitated bacteria in LB and LBCT media were calculated using the following formula: %=(MPN/TCC)X100. Percentages of resuscitated bacteria in P2-P3 samples were below 0.1. (D, E) light microscopy of P1 and P2 samples. Scale bar is 1 μm. Data are presented as the mean ± SEM (n=3).

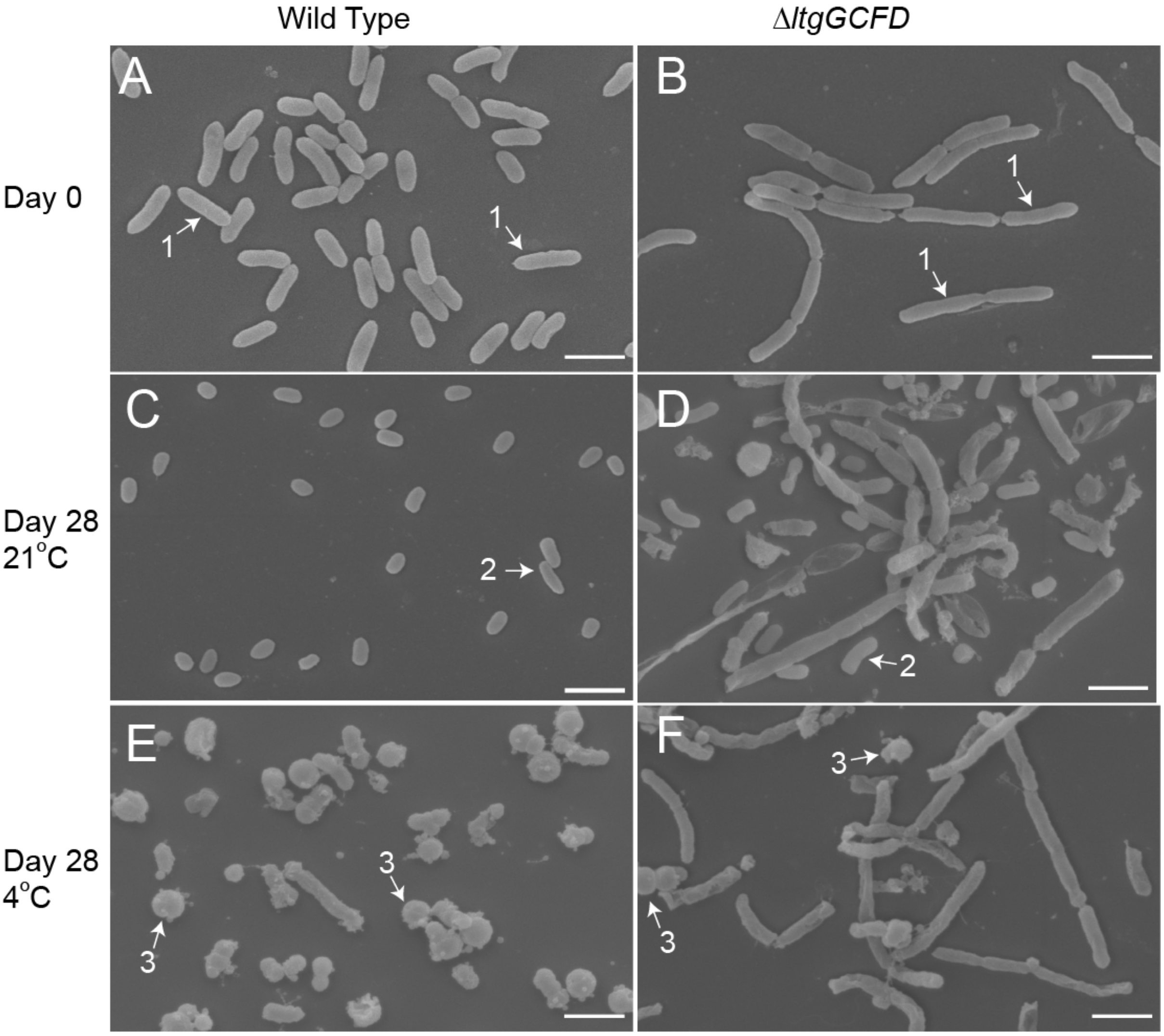

Figure S4. Scanning electron microscopy of starved *B. pseudomallei* wild type (left) and Δ*ltgGCFD* (right). Day 0 (panels A and B) and following 28 days of incubation at 21°C (panels C and D) and 4°C (panels E and F). Panels A, C and E show representative images of starved wild type bacteria, panels B, D and F show representative images of starved Δ*ltgGCFD* bacteria. Arrow 1 shows a rod from a growing culture, arrow 2 indicates shortened rods from starved cultures, arrow 3 shows coccoid cells. SEM scale bars are 2μm.
